# Acyclovir Suppression to Prevent Clinical Recurrences at Delivery After First Episode Genital Herpes in Pregnancy: An Open-Label Trial

**DOI:** 10.1155/S106474490100014X

**Published:** 2001

**Authors:** L. Laurie Scott, Lisa M. Hollier, Donald McIntire, Pablo J. Sanchez, Gregory L. Jackson, George D. Wendel

**Affiliations:** 1 Department of Obstetrics and Gynecology University of Texas Southwestern Medical Center TX USA; 2 Department of Pediatrics University of Texas Southwestern Medical Center Dallas TX USA; 3 580 NW 108th Avenue Plantation FL 33317 USA

## Abstract

*Objective:* To continue evaluation of the use of acyclovir suppression in late pregnancy after first episode genital herpes simplex virus (HSV) infection, using an open-label study design.

*Methods:* Ninety-six women diagnosed with genital herpes for the first time in the index pregnancy were prescribed suppressive acyclovir 400 mg orally three times daily from 36 weeks until delivery in an open-label fashion. Herpes cultures were obtained when patients presented for delivery. Vaginal delivery was permitted if no clinical recurrence was present; otherwise a Cesarean delivery was performed. NeonatalHSV cultures were obtained and infants were followed clinically. Rates of clinical and asymptomatic genital herpes recurrences and Cesarean delivery for genital herpes were measured, and 95% confidence intervals were calculated.

*Results:* In 82 patients (85%) compliant with therapy, only 1% had clinical HSV recurrences at delivery. In an intent to treat analysis of the entire cohort, 4% had clinical recurrences (compared with 18–37% in historical controls). Asymptomatic shedding occurred in 1% of women without lesions at delivery. Two of the four clinical recurrences were HSV-culture positive. No significant maternal or fetal side-effects were observed.

*Conclusions:* In clinical practice the majority of patients are compliant with acyclovir suppression at term. The therapy appears to be effective at reducing clinical recurrences after a first episode of genital herpes complicating a pregnancy.


Infect Dis Obstet Gynecol 2001;9:75–80

### Acyclovir suppression to prevent clinical recurrences at delivery after first episode genital herpes in pregnancy: an open-label trial

**L. Laurie Scott[1], Lisa M. Hollier[1], Donald McIntire[1], Pablo J. Sanchez[2], Gregory L. Jackson[2] and George D. Wendel Jr[1]**

[1]*Department of Obstetrics and Gynecology, and* [2]*Department of Pediatrics, University of Texas Southwestern Medical Center, Dallas, TX*

***Objective:*** To continue evaluation of the use of acyclovir suppression in late pregnancy after first episode genital herpes simplex virus (HSV) infection, using an open-label study design.

***Methods:*** Ninety-six women diagnosed with genital herpes for the first time in the index pregnancy were prescribed suppressive acyclovir 400 mg orally three times daily from 36 weeks until delivery in an open-label fashion. Herpes cultures were obtained when patients presented for delivery. Vaginal delivery was permitted if no clinical recurrence was present; otherwise a Cesarean delivery was performed. Neonatal HSV cultures were obtained and infants were followed clinically. Rates of clinical and asymptomatic genital herpes recurrences and Cesarean delivery for genital herpes were measured, and 95% confidence intervals were calculated.

***Results:*** In 82 patients (85%) compliant with therapy, only 1% had clinical HSV recurrences at delivery. In an intent to treat analysis of the entire cohort, 4% had clinical recurrences (compared with 18–37% in historical controls). Asymptomatic shedding occurred in 1% of women without lesions at delivery. Two of the four clinical recurrences were HSV-culture positive. No significant maternal or fetal side-effects were observed.

***Conclusions:*** In clinical practice the majority of patients are compliant with acyclovir suppression at term. The therapy appears to be effective at reducing clinical recurrences after a first episode of genital herpes complicating a pregnancy.

Key words: Antiviral Agents, Sexually Transmitted Disease, Pregnancy Complications, Infectious

As many as 5% of women have a history of genital herpes and are at risk for transmitting herpes simplex virus (HSV) to their infants during delivery, should they have a peripartum recurrence[1]. Many of these women will experience their first episode of genital herpes while they are pregnant, and they have a high risk of recurrence at the time of delivery[2,3]. To avoid intrapartum HSV exposure and neonatal infection, it is currently recommended that pregnant women with visible genital herpes lesions or prodromal symptoms at the time of labor undergo Cesarean delivery. Gravidas without visible lesions or prodromal symptoms should be allowed to continue in labor, because they have a low risk of neonatal HSV transmission[4]. Using these guidelines, it is estimated that one poor neonatal outcome from HSV infection is averted (at a cost of 2.5 million dollars) for every 1580 Cesarean deliveries performed for maternal clinical HSV recurrences[5].

Correspondence to: L. Laurie Scott, MD, 580 NW 108th Avenue, Plantation, FL 33317

We previously reported a randomized, controlled trial that compared acyclovir suppression with placebo in the last several weeks of pregnancy. We asked whether acyclovir treatment could decrease the incidence of clinical herpes recurrences and thereby decrease the need for cesarean section for this indication[2]. In compliant patients none of the acyclovir-treated ones had a recurrence at the time of delivery, compared with 36% of the placebo-treated patients. In an intent to treat analysis, 8% of the acyclovir-treated patients, and 34% of the placebo-treated patients, had a recurrence at delivery. The differences between treatment groups in both analyses were statistically significant, although the number of patients enrolled in the initial study was relatively small ($n = 46$). The purpose of the present study was to extend our investigation of the effect of suppressive acyclovir on recurrent genital herpes after a first episode infection in pregnancy, using an open-label study design.

### PATIENTS AND METHODS

Eligible patients included any gravida whose first clinical episode of genital herpes occurred during the index pregnancy. All denied a prior diagnosis of herpes, or having had similar lesions before becoming pregnant. The diagnosis was confirmed by a positive viral culture for herpes simplex virus, but viral typing was not performed. Women with primary infection, women with first genital, nonprimary episodes, and women with a first clinical episode of recurrent disease were all eligible[6]. Serology was not performed to distinguish between these groups of patients. Exclusion criteria for enrollment included a serum creatinine level greater than 1.5 mg/dl, immunosuppressive diseases (e.g., human immunodeficiency virus infection), a known requirement for Cesarean delivery (e.g., previous Cesarean with a classical uterine incision), enrollment in another study protocol, a gestational age of greater than 36 weeks upon identification, delivery before 36 weeks' gestation or previous acyclovir intolerance. Subjects were excluded from evaluation of efficacy for voluntary withdrawal, noncompliance with the study protocol, loss of follow-up for delivery, or delivery within one week of beginning treatment. A patient was considered to be noncompliant if she had not taken any study medication for 7 or more days prior to delivery. An intent to treat analysis was performed, which included all patients with known outcomes. Informed consent was obtained from eligible patients by 36 completed weeks of gestation. At 36 weeks' estimated gestational age, patients were prescribed acyclovir 400 mg orally, three times daily until delivery. The women were evaluated weekly after 36 weeks' gestation and were questioned at each visit about genital lesions, prodromal symptoms, compliance, and side-effects from the treatment. An examination for genital HSV lesions was performed at each visit. HSV cultures were obtained only if the patient described prodromal symptoms or if a lesion was identified. The collection, handling, and culture techniques have been described previously[2,7].

Upon presentation for delivery the patients were examined for genital lesions and questioned regarding prodromal symptoms. Herpes cultures of the usual lesion site and cervix were obtained to detect asymptomatic viral shedding. Asymptomatic shedding was defined as a positive maternal HSV culture in the absence of prodromal symptoms or visible lesions. Any visible genital or cervical lesions were also cultured for HSV and classified as clinical recurrences. Obstetric housestaff or nurse practitioners who had been instructed in proper viral culture collection techniques obtained the samples from patients upon admission to the labor and delivery unit. The acyclovir was not continued intrapartum. Patients underwent Cesarean delivery if prodromal symptoms or lesions suspicious for genital herpes were present, regardless of the length of time the amniotic membranes had been ruptured. They were allowed to labor if prodromal symptoms and lesions were absent.

The pediatricians were informed of the mother's participation in the study and a physical exam was performed upon the infant's admission to the newborn nursery. Neonatal HSV cultures were taken from the conjunctiva, oropharynx, and rectum 24–72 hours after delivery. The infants were observed for clinical evidence of acyclovir toxicity (abnormal renal, liver or central nervous system function) and HSV infection. Prophylactic acyclovir therapy was not administered unless the infant was exposed to an active herpes lesion; treatment was left to the discretion of the attending pediatrician. Mothers were instructed to contact the investigators for any problems in the first month after discharge and the infants' medical records were reviewed at least 1 month after delivery.

Apart from the fact that patients were treated in an open-label fashion, this study protocol was the same as the one used in our earlier randomized clinical trial[2]. The primary outcome measured was the frequency of clinical genital herpes recurrences at delivery. Secondary outcomes were the rate of asymptomatic and total HSV detection by culture and the frequency of cesarean delivery for genital herpes. Rates and exact 95% confidence intervals were calculated. The Food and Drug Administration granted an Investigational Drug Number for this protocol, and the investigational review boards of the University of Texas Southwestern Medical Center, Parkland Health and Hospital System and St Paul Medical Center granted approval for the study.

### RESULTS

Ninety-nine patients with a diagnosis of first episode genital herpes during pregnancy were eligible and agreed to participate in the open-label trial. Seventeen women began but did not complete the study protocol, of whom 3 were lost to follow-up. These 14 women were excluded from the evaluation of the efficacy of the therapeutic regimen, but all 96 patients with a known outcome were included in the intent to treat analysis[8,9].

The mean gestational age at diagnosis of the first herpes outbreak was 21.1 weeks. The mean total number of herpes outbreaks (including the initial outbreak) during the pregnancies was 1.3. Other maternal characteristics are provided in Table 1. Eighty-two patients completed the study protocol. One (1.2%) patient had clinically-evident genital herpes recurrence at delivery and required a Cesarean for HSV. Her HSV culture was negative. Asymptomatic shedding was documented in 1/70 (1.4%) patients who had a herpes culture at presentation for delivery (Table 2). The 14 women with known outcomes who were not evaluable were analyzed. Six women delivered within 1 week of starting prophylaxis, five withdrew from the study and three women were noncompliant. Three of these women (21%) had clinical recurrences at delivery, and two of the three (67%) were HSV-culture positive. Eight of the other 11 women had HSV cultures performed at delivery, and all were negative.

The 82 women who completed the trial were combined with the 14 women who were not evaluable for efficacy. In this intent to treat analysis, four of 96 (4.2%) of the women had a clinical

**Table 1** Maternal and neonatal characteristics; data presented as percentage, mean, or median of the sample

| *Characteristic* | *Patients* (n = 99) |
|---|---|
| Ethnicity | |
| Black | 63% |
| White | 7% |
| Hispanic | 30% |
| Age | 21.9 years |
| Parity | 1 |
| Gestational age at diagnosis | 21.1 weeks |
| Outbreaks during pregnancy* | 1.3 |
| Gestational age at delivery* | 39.1 weeks |
| Birthweight* | 3279 g |
| 5-minute Apgar score* | 9 |
| Cord pH* | 7.17 |

*n used in calculations may be different owing to unavailability of information for some patients

**Table 2** Genital herpes recurrences at delivery

| | *Recurrences at delivery* | *Exact 95% confidence interval* |
|---|---|---|
| Efficacy analysis | | |
| Symptomatic recurrences | 1/82 (1.2%) | 0.03–6.61 |
| Asymptomatic shedding | 1/70 (1.4%) | 0.04–7.70 |
| Total HSV detection (symptomatic + asymptomatic) | 1/71 (1.4%) | 0.04–7.60 |
| Intent to treat analysis | | |
| Symptomatic recurrences | 4/96 (4.2%) | 1.15–10.33 |
| Asymptomatic shedding | 1/78 (1.3%) | 0.03–6.94 |
| Total HSV detection (symptomatic + asymptomatic) | 3/82 (3.7%) | 0.76–10.32 |

HSV, herpes simplex virus recurrence of genital herpes at labor and underwent Cesarean delivery. Two of the four women (50%) with clinical recurrences at delivery had positive genital HSV cultures. Asymptomatic HSV shedding occurred in 1.3% (1/78) (Table 2).

The mean gestational age at delivery was 39.1 weeks and the mean birthweight was 3279 g. All but one infant had 5-minute Apgar scores greater than 7. One infant received prophylactic antiviral therapy for 3 days. This neonate was from a noncompliant mother who had prolonged ruptured membranes in the presence of a genital herpes lesion and underwent Cesarean delivery. The infant's HSV cultures were negative, and he ultimately did well. Although no infant had clinical evidence of neonatal herpes, one did have a positive HSV culture when tested at less than 24 hours after delivery, a breach of the study protocol. The mother was clinically asymptomatic, and the maternal HSV cultures obtained at presentation for delivery were negative. The infant was observed without treatment. Repeat herpes cultures obtained at the appropriate time, between 24 and 72 hours of life, were negative. No infant experienced any clinically apparent neurological, hepatic, or renal complications or other adverse effects attributable to acyclovir during the neonatal period.

### DISCUSSION

Historically, women with a first episode genital herpes outbreak in pregnancy have a high rate of recurrence (18–37%) at delivery[2,3,10]. In an effort to prevent intrapartum herpes virus transmission, obstetricians generally resort to Cesarean delivery when patients present for delivery with recurrent genital HSV lesions or prodromal symptoms. Under current guidelines a vaginal delivery is appropriate in the absence of these findings[1,4]. Daily acyclovir therapy suppresses recurrent genital HSV outbreaks in the nonpregnant adult[11], and this treatment has been shown to help prevent herpes reactivation at delivery[2,11,12]. Our study provides further evidence that acyclovir suppressive therapy in the last several weeks of pregnancy decreases genital herpes recurrences at the time of delivery in women whose first genital herpes episode occurred during that pregnancy. However, acyclovir suppression does not completely eliminate the possibility of clinical reactivations, asymptomatic shedding, or even neonatal exposure to HSV.

A theoretical concern has been whether symptomatic recurrences would be converted to asymptomatic shedding episodes by suppressive treatment, thus potentially increasing an infant's perinatal exposure to HSV through a vaginal delivery. Acyclovir suppression in nonpregnant patients decreases asymptomatic shedding by up to 96%[13], however this association has not been evaluated in pregnant women. The reported incidence of asymptomatic HSV shedding at any given time in pregnant women with genital herpes is about 1–2%[14–16]. In the present study suppressive acyclovir therapy decreased the frequency of recurrent HSV lesions without apparently increasing the frequency of asymptomatic viral shedding. These results are reassuring, although they do not demonstrate the same decrease in viral excretion as reported for nonpregnant patients.

On short-term follow-up there were no clinically apparent adverse effects on any of the neonates who had prenatal exposure to acyclovir, 1200 mg daily, at term. Our previous study[2], and those of Stray-Pedersen[12] and Brocklehurst and colleagues[10] do not indicate that any harm is caused by maternal acyclovir suppression. Data from the Acyclovir in Pregnancy Registry do not suggest adverse outcomes attributable to acyclovir in over 1000 prenatal exposures at various gestational ages[17]. Additional anecdotal evidence of acyclovir safety is provided indirectly through reported pediatric clinical experience. Both term and preterm infants treated daily with intravenous acyclovir tolerate the drug well[18,19]. Furthermore, the fetal serum levels in gravidas taking 1200 mg acyclovir daily have been reported to be much lower than the acyclovir levels achieved during neonatal parenteral treatment[18–20]. The lack of adverse effects is reassuring. However, in the absence of large, controlled trials specifically designed to assess neonatal outcomes, including hematologic, renal and hepatic parameters, it cannot yet be concluded that prenatal suppressive acyclovir therapy is completely safe for the fetus. Further evaluation of short- and long-term neonatal outcomes is necessary.

The major limitation to the study is that it was an open-label trial, rather than a double blind, prospective, randomized study. Although the study design is not as rigorous, we felt that it was not ethical to continue the randomization to placebo once the earlier study demonstrated a significant benefit. Other limitations include not identifying HSV type and lack of discrimination between primary, first-episode nonprimary, and first clinically apparent recurrent disease. However, at this time, reliable herpes simplex type-specific serology was not available outside reference laboratories. Our study design reflected the level of information most clinicians would have, that is clinical history and herpes culture results.

When the findings in this cohort are combined with those from our previous randomized trial, the clinical experience with prophylaxis in this group of women with genital herpes is enhanced. Using the evaluable patients in both groups, 1/103 (1.03%; 95% CI, 0.02–5.3) had clinical recurrences. Using all the treated patients in both groups, 6/122 (4.9%; 95% CI, 1.83–10.4) had clinical recurrences. These combined frequencies are dramatically and consistently low given the high rate of expected recurrences of 34–36% from our placebo-treated cohort in the previous study[2]. Furthermore, the observed rate of recurrences in patients who stopped taking suppressive therapy in this study (3 of 14 or 21%) and in the combined cohort (5 of 19 or 26%) underscores the high activity of disease in this group. It also emphasizes the increased risks of recurrence associated with noncompliance.

### CONCLUSIONS

This study demonstrates that acyclovir suppressive therapy given from 36 weeks' gestation until delivery prevents recurrent genital HSV at the time of delivery and is associated with a low frequency of cesarean section for recurrent genital herpes in women who experienced their first episode of genital herpes earlier during the pregnancy. This benefit confirms and extends our previous findings outside a randomized trial. Although the small sample size does not provide sufficient power to draw conclusions regarding the impact of acyclovir suppression of asymptomatic viral shedding or fetal outcome, no changes were detected in the rate of asymptomatic HSV shedding at delivery nor were any short-term, adverse neonatal effects detected.

### REFERENCES

1. Prober CG, Corey L, Brown ZA, *et al.* The management of pregnancies complicated by genital infections with herpes simplex virus. *Clin Infect Dis* 1992;15:1031–8
2. Scott LL, Sanchez PJ, Jackson GL, *et al.* Acyclovir suppression to prevent cesarean delivery after first episode genital herpes. *Obstet Gynecol* 1996;87:69–73
3. Frenkel LM, Garratty EM, Shen JP, *et al.* Clinical reactivation of herpes simplex virus type 2 infection in seropositive pregnant women with no history of genital herpes. *Ann Intern Med* 1993;118:414–18
4. American College of Obstetricians and Gynecologists. Management of Herpes in Pregnancy. ACOG Practice Bulletin, Number 8. Washington DC, The American College of Obstetricians and Gynecologists. October, 1999
5. Randolph AG, Washington AE, Prober CG. Cesarean delivery for women presenting with genital herpes lesions. Efficacy, risks, and costs. *JAMA* 1993;270:77–82
6. Hensleigh PA, Andrews WW, Brown Z, *et al.* Genital herpes during pregnancy: inability to distinguish primary and recurrent infections clinically. *Obstet Gynecol* 1997;89:891–5
7. Hankins GD, Cunningham FG, Luby JP, *et al.* Asymptomatic genital excretion of herpes simplex virus during early labor. *Am J Obstet Gynecol* 1984;150:100–1
8. Makuch R, Johnson M. Issues in planning and interpreting active control equivalence studies. *J Clin Epidemiol* 1989;42:503–11
9. Sackett DL, Gent M. Controversy in counting and attributing events in clinical trials. *N Engl J Med* 1983;301:1410–12

10. Brocklehurst P, Kinghorn G, Carney O, *et al.* A randomised placebo controlled trial of suppressive acyclovir in late pregnancy in women with recurrent genital herpes infection. *Br J Obstet Gynaecol* 1998;105:275–80
11. Goldberg LH, Kaufman R, Kurtz TO, *et al.* Long-term suppression of recurrent genital herpes with acyclovir. *Arch Dermatol* 1993;129:582–7
12. Stray-Pedersen B. Acyclovir in late pregnancy to prevent neonatal herpes simplex. *Lancet* 1990;336 (8717):756
13. Wald A, Zeh J, Barnum G, *et al.* Suppression of subclinical shedding of herpes simplex virus type 2 with acyclovir. *Ann Intern Med* 1996;124:8–15
14. Arvin AM, Hensleigh PA, Prober CG, *et al.* Failure of antepartum maternal cultures to predict the infant's risk of exposure to herpes simplex virus at delivery. *N Engl J Med* 1986;315:796–800
15. Wittek AE, Yeager AS, Au DS, Hensleigh PA. Asymptomatic shedding of herpes simplex virus from the cervix and lesion site during pregnancy. Correlation of antepartum shedding at delivery. *Am J Dis Child* 1984;138:439–42
16. Prober CG. Herpetic vaginitis in 1993. *Clin Obstet Gynecol* 1993;36:177–87
17. Reiff-Eldridge R, Heffner CR, Ephross SA, *et al.* Monitoring pregnancy outcomes after prenatal drug exposure through pregnancy registries: a pharmaceutical company commitment. *Am J Obstet Gynecol* 2000;182:159–63
18. Hintz M, Connor JD, Spector SA, *et al.* Neonatal acyclovir pharmacokinetics in patients with herpes virus infections. *Am J Med* 1982;73:210–14
19. Yeager AS. Use of acyclovir in premature and term neonates. *Am J Med* 1982;73:205–9
20. Frenkel LM, Brown ZA, Bryson YJ, *et al.* Pharmacokinetics of acyclovir in the term human pregnancy and neonate. *Am J Obstet Gynecol* 1991; 164:569–76

RECEIVED 12/20/00; ACCEPTED 03/30/01
